# Electroencephalography Alpha Traveling Waves as Early Predictors of Treatment Response in Major Depressive Episodes: Insights from Intermittent Photic Stimulation

**DOI:** 10.3390/biomedicines13041001

**Published:** 2025-04-21

**Authors:** Xiaojing Guo, Haifeng Zhang, Biyu Zeng, Aoling Cai, Junjie Zheng, Jingshuai Zhou, Yongquan Gu, Minya Wu, Guanhui Wu, Li Zhang, Fei Wang

**Affiliations:** 1 The Fourth School of Clinical Medicine, Nanjing Medical University, Nanjing 210096, China; guoxj@njmu.edu.cn; 2 Department of Neurology, The Affiliated Suzhou Hospital of Nanjing Medical University, Suzhou 215000, China; 3 College of Information Science and Engineering, Northeastern University, Shenyang 110000, China; 4 Early Intervention Unit, Department of Psychiatry, The Affiliated Brain Hospital of Nanjing Medical University, Nanjing 210096, China; 5 Functional Brain Imaging Institute of Nanjing Medical University, Nanjing 210096, China; 6 Department of Geriatric Neurology, The Affiliated Brain Hospital of Nanjing Medical University, Nanjing 210096, China; 7 Department of Mental Health, School of Public Health, Nanjing Medical University, Nanjing 210096, China

**Keywords:** electroencephalography, alpha rhythm, major depressive disorder, depression, photic stimulation, brain waves, biomarkers, treatment outcome, neural oscillations

## Abstract

**Background:** Early evaluation of treatment efficacy in adolescents and young adults with major depressive episodes (MDEs) remains a clinical challenge, often delaying timely therapeutic adjustments. Electroencephalography (EEG) alpha traveling waves, particularly those elicited by intermittent photic stimulation (IPS), may serve as biomarkers reflecting neural dynamics. This study aimed to investigate whether IPS-induced alpha traveling waves could predict early treatment outcomes in transitional-aged youth with MDEs. **Methods**: We recorded EEG signals from 119 patients aged 16–24 years at admission, prior to a standardized two-week treatment regimen. IPS was applied using multiple stimulus frequencies, and alpha traveling waves were analyzed in terms of directionality (forward vs. backward) and hemispheric lateralization. **Results**: Alpha traveling wave amplitudes varied across individuals, depending on stimulus frequency and hemisphere. Notably, a higher amplitude of backward alpha traveling waves at 10 Hz IPS in the left hemisphere significantly predicted positive early treatment response. In contrast, forward waves and right hemisphere responses did not show predictive value. **Conclusions**: IPS-induced backward alpha traveling waves in the left hemisphere may represent promising EEG biomarkers for early prediction of treatment efficacy in youth with MDEs. These findings offer a potential neurophysiological tool to support personalized treatment strategies and inform future clinical applications in adolescent and young adult depression.

## 1. Introduction

Major depressive episodes (MDEs) represent a debilitating clinical manifestation commonly associated with mood disorders, notably major depressive disorder (MDD) and bipolar disorder (BD) [1]. Globally, MDEs exhibit a notably high prevalence among transitional-aged youth (aged 16 to 24) [2], a demographic group particularly vulnerable to early-onset depressive symptoms, chronicity, and significant variability in response to treatment [3]. Clinical management in this population is challenging, as evidenced by the limited efficacy and substantial non-response rates—approximately 30–60%—to current first-line treatments [4]. Consequently, developing innovative methods for the early prediction of treatment response is critical, as timely predictions can significantly inform and enhance individualized therapeutic strategies and overall clinical outcomes [5].

Recent technological advances in electroencephalography (EEG) have provided promising avenues for exploring neural dynamics underlying depressive disorders [6]. Among EEG biomarkers, alpha waves have demonstrated significant potential as indicators of treatment response due to their notable sensitivity and specificity [7]. Traditionally, alpha waves are correlated with relaxed wakefulness and sensory inhibition states, establishing their prominence in depression-related neurophysiological research [8]. More recently, a dynamic phenomenon termed alpha traveling waves has garnered attention. These traveling waves are characterized by the systematic propagation of alpha rhythms across cortical regions, reflecting effective neural communication and synchronization [9]. In contrast to conventional static measures of alpha power, alpha traveling waves offer nuanced insights into the neural adaptability and coordination in response to stimuli [10,11,12].

Intermittent photic stimulation (IPS), an EEG paradigm extensively used in clinical neuroscience, provides an optimal experimental context to investigate alpha traveling waves. By delivering rhythmic visual stimuli, IPS reliably elicits adaptive neural responses, notably the generation of traveling alpha waves, facilitating a dynamic assessment of cortical communication [13,14,15,16]. Given these unique properties, IPS emerges as an invaluable methodological tool for examining how alpha traveling waves might predict therapeutic outcomes, particularly in clinical populations experiencing depression.

Furthermore, interhemispheric asymmetry in alpha rhythm power has previously been associated with prognostic implications for depression treatment [17]. Prior studies suggest that treatment-responsive individuals typically exhibit alpha dominance in the left hemisphere, whereas non-responsive individuals display greater alpha activity within the right hemisphere [18,19]. Quantitatively, the sensitivity and specificity of alpha asymmetry in predicting treatment responses have reached approximately 63.6% and 71.4%, respectively [20]. These findings highlight the importance of accounting for hemisphere-specific neural dynamics when evaluating EEG predictors of treatment efficacy.

In light of these insights, the present study aims to explore the role of IPS-induced alpha traveling waves as early predictors of treatment response in transitional-aged youth experiencing MDEs. By investigating these neural dynamics across both hemispheres, this research intends to provide novel neurophysiological markers capable of guiding early-stage therapeutic decision-making. Ultimately, the study aspires to enhance personalized treatment approaches, thereby improving clinical outcomes for individuals coping with major depressive episodes.

## 2. Materials and Methods

This study employed a multimodal approach integrating clinical assessments and EEG to investigate the predictive role of alpha traveling waves in early treatment response among transitional-aged youth experiencing MDEs. Participants underwent standardized psychiatric evaluation and EEG recording under IPS to elicit traveling waves. Signal processing and statistical modeling were performed to quantify wave dynamics and assess their association with clinical outcomes. The methodology was designed based on established neurophysiological research frameworks, with adaptations to suit the study’s objectives.

### 2.1. Participants

Conducted at a single site, the study included 119 transitional-aged youth (mean age = 17.27 ± 1.65 years, range: 16–24 years) diagnosed with mood disorders (MDD or BD, currently in an MDE). Among them, 34 were male (28.57%) and 85 were female (71.43%). Participants were recruited from inpatient services at the Affiliated Brain Hospital of Nanjing Medical University. Ethical approval was obtained from the Ethics Committee of the Affiliated Brain Hospital of Nanjing Medical University (2020-KY029-01). Written informed consent was acquired from participants or their legal guardians (if under 18) following a detailed explanation of the study procedures.

Clinical diagnoses were determined by two trained psychiatrists. For individuals aged 18 or older, the Structured Clinical Interview for DSM-IV-TR Axis I Disorders (SCID) was administered, while those under 18 were assessed using the Schedule for Affective Disorders and Schizophrenia for School-Age Children—Present and Lifetime Version (K-SADS-PL). Depression severity was measured with the 17-item Hamilton Depression Rating Scale (HAMD-17) at baseline and two weeks post-treatment [21,22].

Participants met the following inclusion criteria: (1) DSM-IV-based diagnosis of MDD or BD without other Axis I disorders; (2) currently experiencing a major depressive episode; and (3) baseline HAMD-17 score ≥ 17 [23]. Exclusion criteria were as follows: (1) co-morbid Axis I disorders (e.g., substance or alcohol abuse/dependence); (2) medical conditions affecting brain function (e.g., hypertension, diabetes, cancer); (3) neurological disorders (e.g., epilepsy, head trauma); (4) mood disorders secondary to physical illnesses (e.g., thyroid dysfunction); and (5) contraindications for EEG (e.g., intracranial implants or scalp injuries).

### 2.2. Treatment and Measurement

Following baseline EEG recordings and clinical assessments, participants underwent two weeks of standardized acute treatment in a real-world clinical setting. Medications prescribed by clinicians included mood stabilizers, antipsychotics, and sedative–hypnotics, in accordance with standard clinical guidelines for adolescent mood disorders [24,25]. Medication regimens were determined by clinicians based on individual patient needs without intervention from researchers. Detailed medication information is provided in Table 1.

Early treatment response was assessed by calculating the percentage reduction in HAMD-17 scores after two weeks of treatment using the following formula: ([Baseline HAMD-Post-treatment HAMD]/Baseline HAMD) × 100%.

### 2.3. EEG Experimental Design

**Figure 1:**
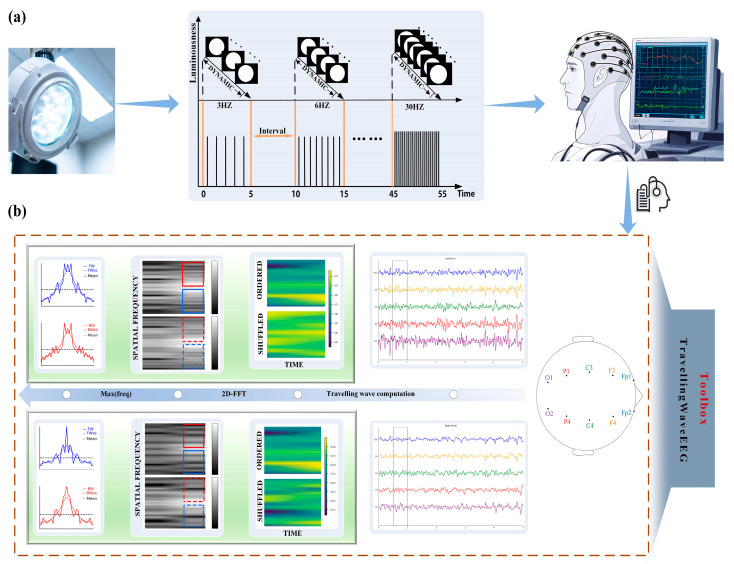
Pipeline of EEG signal acquisition and traveling wave feature extraction. (**a**) Collecting the EEGs of participants at photic stimulus of different frequencies and performing manual preprocessing operations. (**b**) Use of the Travelling-waves-EEG toolbox to extract the propagation intensity of forward and backward waves in the left and right hemispheres from the preprocessed EEG.

EEG recordings were conducted in a quiet, dark, and controlled environment. Participants were seated comfortably, approximately 30 cm from the visual stimulus, remaining awake and relaxed. The experimental protocol (Figure 1a) consisted of two stages: initially, resting-state EEG with closed eyes was recorded for at least 10 s to stabilize signals; subsequently, IPS was applied sequentially at frequencies of 3 Hz, 6 Hz, 10 Hz, 15 Hz, 24 Hz, and 30 Hz. Each frequency was presented for 5 s, followed by a 5 s resting interval. The photic stimulus was administered using a Nihon Kohden LS-703A unit with a xenon lamp, delivering pulses at 1.28 J per pulse (Nihon Kohden Corporation, Tokyo, Japan).

### 2.4. EEG Recording and Pre-Processing

Continuous EEG signals were recorded using an 18-channel Nihon Kohden EEG-1200C system at a sampling rate of 200 Hz (Nihon Kohden Corporation, Tokyo, Japan). Electrodes were placed according to the international 10–20 system, ensuring standardized spatial distribution. The reference electrodes were positioned at the bilateral mastoids (M1 and M2), and the grounding electrode was located at the FPz site. An electro-oculogram (EOG) was not recorded. A band-pass filter (0.5–45 Hz) and a notch filter (48–52 Hz) were applied to minimize noise. Independent component analysis (ICA) was performed to remove artifacts, including eye movements and cardiac activity. Epochs contaminated by excessive noise were identified via visual inspection and excluded from further analysis. Finally, EEG data were re-referenced to the average of all electrodes. Preprocessing was conducted using the EEGLAB 2022 toolbox [26] within MATLAB R2020b.

### 2.5. Traveling Wave Quantification

We specifically focused on alpha-band (8–12 Hz) traveling waves along two hemispheric pathways, namely Fp1-F3-C3-P3-O1 (left hemisphere) and Fp2-F4-C4-P4-O2 (right hemisphere). Traveling wave amplitudes were quantified using the Travelling-waves-EEG toolbox developed by Alamia et al. [9,27]. EEG signals were segmented into overlapping windows (500 ms duration, 250 ms overlap), then mapped into two-dimensional (2D) matrices subjected to a two-dimensional fast Fourier transform (2D-FFT) to extract and quantify the amplitude of forward waves (FWs, propagating from occipital to frontal) and backward waves (BWs, propagating from frontal to occipital).

### 2.6. Statistical Analysis

Statistical analyses comprised two stages. Initially, one-sample t-tests assessed whether amplitudes of forward and backward traveling waves significantly exceeded zero, applying false discovery rate (FDR) correction. Subsequently, a mixed-effects model was employed to evaluate the influences of stimulus frequencies and hemispheric channels on traveling wave amplitudes. Mixed-effects models were chosen due to their capacity to handle repeated measurements and individual variations [28,29,30,31]. Traveling wave amplitude served as the dependent variable, with fixed effects for IPS frequency categories (low: 3, 6 Hz; mid: 10 Hz; high: 15, 24, 30 Hz) and hemispheric channels (left, right). This classification is based on the brain’s natural tendency to produce harmonic or subharmonic oscillations [32,33,34], while individual participant variability was included as a random effect to control for baseline differences.

Second, we analyzed the relationship between traveling wave amplitudes and treatment response, defined by the percentage reduction in HAMD-17 scores. Simple linear regression analyses were first conducted separately for each hemisphere to identify traveling wave features significantly associated with treatment response. These significant wave features were subsequently incorporated into a multiple linear regression model, adjusting for age and gender as covariates, to identify the most influential predictors of treatment efficacy. Separate regression models for each hemisphere were used to better capture potential hemispheric asymmetries in predictive relationships, reducing inter-hemispheric variability and improving interpretability. This methodological approach allowed us to control for potential demographic confounders, thus enhancing the reliability and interpretability of the findings.

## 3. Results

### 3.1. Sociodemographic

Demographic and medication details are provided in Table 1.

### 3.2. Alpha Traveling Wave Analysis

The presence and characteristics of alpha traveling waves were first examined through one-sample *t*-tests, revealing that amplitudes of both alpha forward waves (α-FW) and alpha backward waves (α-BW) were significantly greater than zero across all measured time points (*p* < 0.001), confirming the occurrence of traveling waves in both directions among transitional-aged youth with MDEs.

Subsequently, a mixed-effects model analysis was conducted to investigate the effects of IPS frequency categories (low frequency: 3 Hz, 6 Hz; mid frequency: 10 Hz; high frequency: 15 Hz, 24 Hz, 30 Hz) and hemispheric channels (left and right hemispheres) on traveling wave amplitudes. Results indicated significant main effects of stimulus frequency category on both α-FW and α-BW amplitudes. Specifically, low-frequency and mid-frequency IPS significantly reduced the amplitudes of both α-FW and α-BW compared with high-frequency IPS (low frequency: β = −10.497, *p* < 0.001; mid frequency: β = −21.342, *p* < 0.001), suggesting that lower stimulation frequencies exert stronger modulation effects on alpha traveling waves. High-frequency stimulation (HF IPS) does not appear in Table 2, because it was set as the reference level, meaning that all other frequency coefficients represent changes relative to it. Similarly, the interaction terms (IPS frequency × hemisphere) do not include the high-frequency (HF) IPS condition, as all interaction effects are calculated relative to it.

Regarding hemispheric differences, α-FW amplitudes did not significantly differ between hemispheres (β = 0.534, *p* = 0.294), and no significant interaction between the stimulus frequency category and hemisphere was observed (*p* > 0.05 for all interactions). However, significant hemispheric effects emerged for α-BW amplitude, with greater amplitude observed in the right hemisphere compared to the left (right vs. left: β = 0.919, *p* < 0.05), highlighting notable hemispheric asymmetry in backward alpha propagation.

### 3.3. Relationship Between Alpha Traveling Waves and Treatment Response

In this section, we aim to examine whether alpha traveling wave amplitudes could predict treatment response in transitional-aged youth with MDEs. Clinical treatment effectiveness was quantified using the reduction rate of HAMD-17 scores following two weeks of treatment. Given previous findings indicating significant hemispheric differences in alpha traveling wave amplitudes, particularly highlighting significant hemispheric effects for alpha backward waves (α-BW), we conducted separate analyses for left and right hemispheric channels to clarify their respective predictive values.

#### 3.3.1. Left Hemisphere Channel

Univariate regression analyses in the left hemisphere revealed significant positive correlations between α-BW amplitudes and clinical treatment response across all IPS frequency conditions (Table 3). Specifically, higher α-BW amplitudes at low-frequency IPS (*β* = 0.204, *p* = 0.0261), mid-frequency IPS (*β* = 0.327, *p* = 0.0003), and high-frequency IPS (*β* = 0.227, *p* = 0.0129) were significantly associated with greater reductions in HAMD-17 scores. In contrast, alpha forward wave (α-FW) amplitudes did not significantly correlate with treatment outcomes at any frequency condition. A multivariate regression analysis controlling for age and gender confirmed that only α-BW amplitudes at mid-frequency IPS (10 Hz) remained significantly associated with treatment response (*β* = 0.2743, *p* = 0.018), whereas the relationships observed at low- and high-frequency IPS were no longer significant.

#### 3.3.2. Right Hemisphere Channel

Univariate regression analyses in the right hemisphere demonstrated significant positive correlations between α-BW amplitudes and treatment response for low-frequency IPS (*β* = 0.209, *p* = 0.023) and high-frequency IPS (*β* = 0.225, *p* = 0.014) (Table 4). However, after adjusting for age and gender in multivariate regression analysis, these associations were no longer statistically significant. Additionally, α-FW amplitudes showed no significant correlations with treatment response at any frequency condition. 

#### 3.3.3. Predictive Analysis

**Figure 2:**
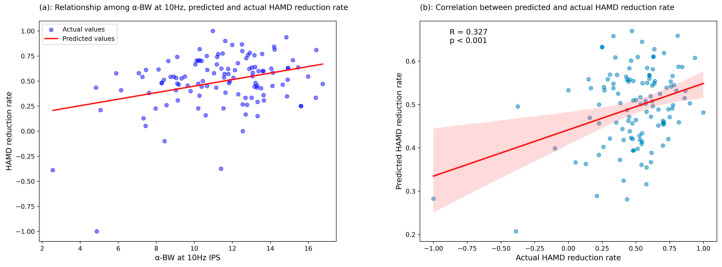
Scatter plot of the correlation between actual and predicted values of HAMD reduction rate based on α-BW at 10 Hz IPS. (**a**) Blue points indicate actual HAMD reduction rates, and the red line represents predictions based on α-BW at 10 Hz IPS. (**b**) Correlation between actual and predicted HAMD reduction rates (*R* = 0.327, *p* < 0.01), with the red regression line indicating the linear relationship. The red shaded area represents the 95% confidence interval of the regression, and the blue dots represent individual data points.

To evaluate the predictive capability of the identified significant predictor, we performed further analysis by entering α-BW amplitudes at mid-frequency IPS (10 Hz, left hemisphere) into a multivariate regression model, adjusting for age and gender, to generate predicted HAMD reduction rates. Correlation analysis between actual and predicted values demonstrated a statistically significant relationship (*R* = 0.327, *p* < 0.01; Figure 2), supporting the predictive validity of α-BW amplitudes at 10 Hz IPS in the left hemisphere for early treatment outcomes.

In summary, α-BW amplitudes elicited by mid-frequency (10 Hz) photic stimulation in the left hemisphere were significantly associated with early treatment response, whereas no such predictive relationship was observed in the right hemisphere. Additionally, α-FW amplitudes did not predict treatment response in either hemisphere across all stimulation frequencies.

## 4. Discussion

Our study provides a significant innovation by examining the lateralization and directionality of alpha traveling waves in response to IPS, which may serve as a potential predictor of early treatment response in MDEs.

Traditional static EEG measures, such as resting-state alpha power and frontal alpha asymmetry [35], have shown limited consistency across studies. While some findings suggest that increased frontal alpha power is associated with better treatment outcomes [36,37], others report the opposite [38,39], leading to ambiguity in their clinical application. To address these inconsistencies, recent research has shifted toward dynamic EEG metrics, which offer richer spatiotemporal insights into brain function [40]. Alpha traveling waves, characterized by systematic phase propagation across cortical regions, have emerged as a novel indicator of neural communication and functional connectivity [41]. Unlike static measures, these waves capture real-time information flow, reflecting cortical adaptability and network efficiency [42,43]. Moreover, they may act as functional bridges between distributed brain networks—an important consideration in MDD, where disrupted connectivity is frequently observed. Additionally, by utilizing IPS, we introduce a non-invasive, cost-effective method for inducing and assessing alpha traveling waves, making it feasible for integration into clinical EEG assessments [44]. Extensive research using this dynamic process has revealed alpha traveling waves as a fundamental feature of cortical activity during visual perception [45,46]. These waves, shaped by neuronal network organization and the balance between excitation and inhibition, reflect the brain’s dynamic functional architecture [43,47,48]. Such mechanisms underlie individual variability in wave patterns, accounting for differences in wave amplitudes observed across participants under identical photic stimulation. That is, IPS only provides new insights into the neurophysiological underpinnings of MDD but also offers a practical framework for incorporating dynamic EEG biomarkers into routine clinical evaluations.

We aim to explain why α-BW induced by 10 Hz IPS predicts early treatment response. From a neurobiological perspective, backward traveling waves are thought to represent top-down predictive signals originating from higher cortical areas, integrating sensory inputs with prior experiences [49,50,51]. Enhanced backward traveling wave activity may reflect stronger neural predictive and adaptive capacities [52], which are critical for emotional regulation and recovery from depressive episodes. This interpretation is supported by findings from Ryan et al., who linked predictive coding deficits with impaired emotional regulation in depression [53]. In contrast, forward traveling waves, primarily associated with bottom-up error correction processes [51], did not significantly predict treatment response. This aligns with predictive coding theory, suggesting that therapeutic effectiveness in depression may depend more on top-down prediction mechanisms rather than error correction processes alone.

The left hemispheric dominance in predictive α-BW activity may reflect functional specialization related to emotional processing and adaptive neural mechanisms. Prior studies have demonstrated hemispheric differences in temporal dynamics and sensory integration [54,55], further supporting our findings. These inter-hemispheric differences may explain why left hemisphere α-BW, rather than right hemisphere activity, emerged as a significant predictor of clinical outcomes in our sample. Additionally, our observation of hemispheric asymmetry in predictive α-BW aligns with EEG and neuroimaging research showing lateralized cortical activity patterns linked to emotional and cognitive processing in depression. Notably, our identification of left hemispheric specificity contrasts with prior studies that have not emphasized hemispheric differences in EEG predictors of treatment response, underscoring the novelty and clinical relevance of our findings. This left hemisphere specificity may reflect intrinsic neurobiological asymmetries, particularly in frontal–parietal networks associated with emotional regulation, decision-making, and reward processing [56]. Such asymmetry may help explain differential treatment outcomes by highlighting hemisphere-specific neural adaptations critical for therapeutic efficacy.

Furthermore, the specificity of the 10 Hz IPS condition highlights the significance of alpha oscillations at this frequency, which are strongly associated with sensory integration, neural synchronization, and predictive processing. Van Rullen’s “perceptual echo” hypothesis further supports this notion, identifying 10 Hz as an optimal frequency for eliciting stable brain responses [57]. In contrast, α-BW amplitudes induced by lower and higher IPS frequencies did not exhibit robust predictive associations, likely due to their distinct neurophysiological roles [16,58]. Low-frequency stimulation predominantly influences resting-state synchronization [54], while high-frequency stimulation modulates neural discrimination processes [59]. This frequency-dependent effect reinforces the idea that 10 Hz IPS optimally engages predictive neural mechanisms relevant to depression treatment outcomes.

Clinically, our findings suggest that α-BW amplitudes elicited by 10 Hz IPS in the left hemisphere may hold promise as potential predictors of treatment response. Incorporating these EEG biomarkers into routine clinical practice could enhance personalized treatment strategies, allowing for early adjustments and potentially improved therapeutic outcomes. By providing clinicians with a reliable objective indicator of neural responsiveness, EEG-based biomarkers may reduce the time and resource investment required for identifying effective treatments.

Despite these promising findings, certain limitations must be considered. First, as a real-world study, treatment regimens varied among participants, and while baseline EEG and clinical assessments were conducted before any new treatment adjustments, prior medication exposure cannot be entirely ruled out. Future studies should consider stratifying participants by medication history or implementing statistical controls for treatment effects. Second, the study involved a relatively homogeneous sample, requiring replication in larger multi-center cohorts to enhance generalizability. Additionally, the predictive validity of α-BW biomarkers was assessed over a short two-week period; future research should examine their stability over longer durations and across different treatment modalities. Finally, integrating other neuroimaging techniques, such as functional MRI in combination with high-density EEG, could provide deeper mechanistic insights and further validate EEG-based biomarkers for personalized psychiatric care.

## 5. Conclusions

This study advances our understanding of EEG alpha dynamics as biomarkers for depression treatment response. The identification of IPS-induced alpha backward traveling waves within the left hemisphere as predictors of early clinical outcomes represents a significant step forward in developing objective neural-based markers for personalized psychiatric care. These findings not only enhance theoretical insights into EEG-based neurophysiological mechanisms but also hold promise for integration into clinical decision-making. While further validation across diverse populations and extended treatment durations is necessary, our results highlight the clinical relevance and transformative potential of dynamic EEG analyses in mental health research. Future studies should focus on optimizing analytical frameworks, incorporating multimodal neuroimaging approaches, and exploring the longitudinal stability of these biomarkers to refine predictive accuracy and clinical applicability.

## Figures and Tables

**Table 1:** Sociodemographic characteristics and medication information of all patients.

Characteristics	All (*n* = 119)
Age at EEG (year)	17.27 ± 1.65
Gender	
Male (%)	34 (28.57%)
Female (%)	85 (71.43%)
Years of education	10.86 ± 2.01
Pre-treatment HAMD	24.18 ± 5.62
2-week-treatment HAMD	11.70 ± 5.22
Medication	
Mood stabilizer (%)	95 (79.83%)
Antipsychotics (%)	112 (94.12%)
Sedative–hypnotic (%)	62 (52.10%)

Data are presented as either *n* (%) or means ± standard deviations. HAMD: Hamilton Depression Rating Scale.

**Table 2:** Mixed-effects model results for alpha forward and backward traveling waves.

Variables	Coefficient	Standard Error	*z*-Statistic	*p*-Value	95% CI
**Alpha Forward Waves (α-FW)**					
Intercept	31.965	0.468	68.350	**<0.001**	31.048~32.881
LF IPS	−10.497	0.509	−20.636	**<0.001**	−11.494~−9.500
MF IPS	−21.342	0.509	−41.956	**<0.001**	−22.339~−20.345
Right vs. Left Channel	0.534	0.509	1.050	0.294	−0.463~1.531
LF IPS ×Channel Interaction	−0.985	0.719	−1.370	0.171	−2.395~0.425
MF IPS × Channel Interaction	−0.258	0.719	−0.358	0.720	−1.668~1.152
Group Variance	10.630	0.480			
**Alpha Backward Waves (α-BW)**					
Intercept	33.520	0.402	83.304	**<0.001**	32.731~34.309
LF IPS	−10.753	0.445	−24.145	**<0.001**	−11.626~−9.881
MF IPS	−22.144	0.445	−49.721	**<0.001**	−23.017~−21.271
Right vs. Left Channel	0.919	0.445	2.065	**0.039**	0.047~1.792
LF IPS × Channel Interaction	−0.264	0.630	−0.420	0.675	−1.499~0.970
MF IPS × Channel Interaction	−0.851	0.630	−1.352	0.176	−2.086~0.383
Group Variance	7.466	0.392			

Note: LF: low frequency; MF: mid frequency; CI: confidence interval. Significant correlations (*p* < 0.05) are indicated in bold.

**Table 3:** Regression analyses of alpha traveling wave amplitudes in the left hemisphere and early treatment response in transitional-aged youth.

Variables	Univariate			Multivariate		
	Standardized *β* (95% CI for *β*)	*t*-Value	*p*-Value	Standardized *β* (95% CI for *β*)	*t*-Value	*p*-Value
Age	−0.179 (−0.360, 0.001)	−1.968	0.051	−0.167 (−0.341, 0.007)	−1.899	0.060
Gender	−0.034 (−0.217, 0.149)	−0.368	0.714	−0.040 (−0.214, 0.135)	−0.449	0.654
α-BW at LF IPS	0.204 (0.025, 0.383)	2.253	**0.026**	−0.016 (−0.254, 0.222)	−0.131	0.896
α-BW at MF IPS	0.327 (0.154, 0.500)	3.743	**<0.001**	0.274 (0.048, 0.500)	2.405	**0.018**
α-BW at HF IPS	0.227 (0.049, 0.406)	2.525	**0.013**	0.090 (−0.146, 0.327)	0.757	0.451
α-FW at LF IPS	0.108 (−0.074, −0.290)	1.178	0.241	-	-	-
α-FW at MF IPS	0.072 (−0.111, 0.254)	0.778	0.438	-	-	-
α-FW at HF IPS	0.066 (−0.117, 0.248,)	0.711	0.479	-	-	-

Note: LF: low frequency; MF: mid frequency; HF: high frequency; CI: confidence interval. Significant correlations (*p* < 0.05) are highlighted in bold.

**Table 4:** Regression analyses of alpha traveling wave amplitudes in the right hemisphere and early treatment response in transitional-aged youth.

Variables	Univariate			Multivariate		
	Standardized *β* (95% CI for *β*)	*t*-Value	*p*-Value	Standardized *β* (95% CI for *β*)	*t*-Value	*p*-Value
Age	−0.179 (−0.360, 0.001)	−1.968	0.051	−0.188 (−0.367, −0.009)	−2.082	0.040
Gender	−0.034 (−0.217, 0.149)	−0.368	0.714	−0.047 (−0.224, 0.130)	−0.524	0.601
α-BW at LF IPS	0.209 (0.030, 0.388)	2.311	**0.023**	0.074 (−0.155, 0.304)	0.642	0.522
α-BW at MF IPS	0.130 (−0.051, 0.312)	1.419	0.159	-	-	-
α-BW at HF IPS	0.225 (0.047, 0.404)	2.499	**0.014**	0.193 (−0.037, 0.423)	1.664	0.099
α-FW at LF IPS	0.067 (−0.116, 0.250)	0.727	0.469	-	-	-
α-FW at MF IPS	0.158 (−0.023, 0.339)	1.733	0.086	-	-	-
α-FW at HF IPS	0.127 (−0.055, 0.308)	1.380	0.170	-	-	-

Note: LF: low frequency; MF: mid frequency; HF: high frequency; CI: confidence interval. Significant correlations (*p* < 0.05) are highlighted in bold.

## Data Availability

Data are available from the authors upon request.

## Abbreviations

The following abbreviations are used in this manuscript:

EEG	Electroencephalography
MDE	Major depressive episode
IPS	Intermittent photic stimulation
BD	Bipolar disorder
MDD	Major depressive disorder
HAMD-17	The 17-item Hamilton Depression Rating Scale
α-FW	Alpha forward waves
α-BW	Alpha backward waves

## References

[1] Nesvåg R., Bramness J.G., Handal M., Hartz I., Hjellvik V., Skurtveit S. (2018). The Incidence, Psychiatric Co-Morbidity and Pharmacological Treatment of Severe Mental Disorders in Children and Adolescents. Eur. Psychiatr..

[2] Wong S.M.Y., Chen E.Y.H., Suen Y.N., Wong C.S.M., Chang W.C., Chan S.K.W., McGorry P.D., Morgan C., van Os J., McDaid D. (2023). Prevalence, Time Trends, and Correlates of Major Depressive Episode and Other Psychiatric Conditions among Young People amid Major Social Unrest and COVID-19 in Hong Kong: A Representative Epidemiological Study from 2019 to 2022. Lancet Reg. Health—West. Pac..

[3] KSABTG. APA Official Actions Position Statement on Transitional Aged Youth. American Psychiatric Association, 2024. www.psychiatry.org/getattachment/6e0f309e-76b6-43f1-8c87-e1d1c986d2b6/Position-Transitional-Aged-Youth.pd.

[4] Dwyer J.B., Stringaris A., Brent D.A., Bloch M.H. (2020). Annual Research Review: Defining and Treating Pediatric Treatment-resistant Depression. Child. Psychol. Psychiatry.

[5] Iznak A.F., Iznak E.V. (2022). EEG Predictors of Therapeutic Responses in Psychiatry. Neurosci. Behav. Physi.

[6] Simmatis L., Russo E.E., Geraci J., Harmsen I.E., Samuel N. (2023). Technical and Clinical Considerations for Electroencephalography-Based Biomarkers for Major Depressive Disorder. Npj Ment. Health Res..

[7] Simon L., Blay M., Galvao F., Brunelin J. (2021). Using EEG to Predict Clinical Response to Electroconvulsive Therapy in Patients With Major Depression: A Comprehensive Review. Front. Psychiatry.

[8] Zhang H., Watrous A.J., Patel A., Jacobs J. (2018). Theta and Alpha Oscillations Are Traveling Waves in the Human Neocortex. Neuron.

[9] Alamia A., VanRullen R. (2019). Alpha Oscillations and Traveling Waves: Signatures of Predictive Coding?. PLoS Biol..

[10] Sponheim S.R., Stim J.J., Engel S.A., Pokorny V.J. (2023). Slowed Alpha Oscillations and Percept Formation in Psychotic Psychopathology. Front. Psychol..

[11] Ippolito G., Bertaccini R., Tarasi L., Di Gregorio F., Trajkovic J., Battaglia S., Romei V. (2022). The Role of Alpha Oscillations among the Main Neuropsychiatric Disorders in the Adult and Developing Human Brain: Evidence from the Last 10 Years of Research. Biomedicines.

[12] Miljevic A., Bailey N.W., Murphy O.W., Perera M.P.N., Fitzgerald P.B. (2023). Alterations in EEG Functional Connectivity in Individuals with Depression: A Systematic Review. J. Affect. Disord..

[13] Vranic-Peters M., O’Brien P., Seneviratne U., Reynolds A., Lai A., Grayden D.B., Cook M.J., Peterson A.D.H. (2024). Response to Photic Stimulation as a Measure of Cortical Excitability in Epilepsy Patients. Front. Neurosci..

[14] Oppermann H., Haueisen J. Transient Events during Photic Driving in Single-Trial EEG within the Second Harmonics. Proceedings of the 2024 46th Annual International Conference of the IEEE Engineering in Medicine and Biology Society (EMBC).

[15] Oppermann H., Thelen A., Haueisen J. (2024). Single-Trial EEG Analysis Reveals Burst Structure during Photic Driving. Clin. Neurophysiol..

[16] Tsoneva T., Garcia-Molina G., Desain P. (2015). Neural Dynamics during Repetitive Visual Stimulation. J. Neural Eng..

[17] Fitzgerald P.J. (2024). Frontal Alpha Asymmetry and Its Modulation by Monoaminergic Neurotransmitters in Depression. Clin. Psychopharmacol. Neurosci..

[18] Jaworska N., Blier P., Fusee W., Knott V. (2012). Alpha power, alpha asymmetry and anterior cingulate cortex activity in depressed males and females. J. Psychiatr. Res..

[19] Baskaran A., Farzan F., Milev R., Brenner C.A., Alturi S., Pat McAndrews M., Blier P., Evans K., Foster J.A., Frey B.N. (2017). The Comparative Effectiveness of Electroencephalographic Indices in Predicting Response to Escitalopram Therapy in Depression: A Pilot Study. J. Affect. Disord..

[20] Bruder G.E., Sedoruk J.P., Stewart J.W., McGrath P.J., Quitkin F.M., Tenke C.E. (2007). Electroencephalographic Alpha Measures Predict Therapeutic Response to a Selective Serotonin Reuptake Inhibitor Antidepressant: Pre- and Post-Treatment Findings. Biol. Psychiatry.

[21] Hamilton M. (1960). A Rating Scale For Depression. J. Neurol. Neurosurg. Psychiatry.

[22] Rost N., Binder E.B., Brückl T.M. (2022). Predicting Treatment Outcome in Depression: An Introduction into Current Concepts and Challenges. Eur. Arch. Psychiatry Clin. Neurosci..

[23] Zimmerman M., Martinez J.H., Young D., Chelminski I., Dalrymple K. (2013). Severity Classification on the Hamilton Depression Rating Scale. J. Affect. Disord..

[24] Tournier M., Neumann A., Pambrun E., Weill A., Chaffiol J.-P., Alla F., Bégaud B., Maura G., Verdoux H. (2019). Conventional Mood Stabilizers and/or Second-Generation Antipsychotic Drugs in Bipolar Disorders: A Population-Based Comparison of Risk of Treatment Failure. J. Affect. Disord..

[25] Dwyer J.B., Landeros-Weisenberger A., Johnson J.A., Londono Tobon A., Flores J.M., Nasir M., Couloures K., Sanacora G., Bloch M.H. (2021). Efficacy of Intravenous Ketamine in Adolescent Treatment-Resistant Depression: A Randomized Midazolam-Controlled Trial. Am. J. Psychiatry.

[26] Delorme A., Makeig S. (2004). EEGLAB: An Open Source Toolbox for Analysis of Single-Trial EEG Dynamics Including Independent Component Analysis. J. Neurosci. Methods.

[27] Alamia A., Terral L., D’ambra M.R., VanRullen R. (2023). Distinct. Roles of Forward and Backward Alpha-Band. Waves in Spatial Visual Attention. Elife.

[28] Baayen R.H., Davidson D.J., Bates D.M. (2008). Mixed-Effects Modeling with Crossed Random Effects for Subjects and Items. J. Mem. Lang..

[29] Frömer R., Maier M., Abdel Rahman R. (2018). Group-Level EEG-Processing Pipeline for Flexible Single Trial-Based Analyses Including Linear Mixed Models. Front. Neurosci..

[30] Huang Y., Erdogmus D., Pavel M., Mathan S. (2008). Mixed Effects Models for EEG Evoked Response Detection. Proceedings of the 2008 IEEE Workshop on Machine Learning for Signal Processing.

[31] Riha C., Güntensperger D., Kleinjung T., Meyer M. (2020). Accounting for Heterogeneity: Mixed-Effects Models in Resting-State EEG Data in a Sample of Tinnitus Sufferers. Brain Topogr..

[32] Regan D. (1966). Some Characteristics of Average Steady-State and Transient Responses Evoked by Modulated Light. Electroencephalogr. Clin. Neurophysiol..

[33] Gu M., Pei W., Gao X., Wang Y. (2024). An Open Dataset for Human SSVEPs in the Frequency Range of 1-60 Hz. Sci. Data.

[34] Srinivasan R., Bibi F.A., Nunez P.L. (2006). Steady-State Visual Evoked Potentials: Distributed Local Sources and Wave-Like Dynamics Are Sensitive to Flicker Frequency. Brain Topogr..

[35] Van der Vinne N., Vollebregt M.A., van Putten M.J.A.M., Arns M. (2017). Frontal alpha asymmetry as a diagnostic marker in depression: Fact or fiction? A meta-analysis. NeuroImage Clin..

[36] Lee P.F., Kan D.P.X., Croarkin P., Phang C.K., Doruk D. (2018). Neurophysiological Correlates of Depressive Symptoms in Young Adults: A Quantitative EEG Study. J. Clin. Neurosci..

[37] Hosseinifard B., Moradi M.H., Rostami R. (2013). Classifying Depression Patients and Normal Subjects Using Machine Learning Techniques and Nonlinear Features from EEG Signal. Comput. Methods Programs Biomed..

[38] Cai H., Sha X., Han X., Wei S., Hu B. Pervasive EEG Diagnosis of Depression Using Deep Belief Network with Three-Electrodes EEG Collector. Proceedings of the 2016 IEEE International Conference on Bioinformatics and Biomedicine (BIBM).

[39] Shen J., Zhao S., Yao Y., Wang Y., Feng L. A Novel Depression Detection Method Based on Pervasive EEG and EEG Splitting Criterion. Proceedings of the 2017 IEEE International Conference on Bioinformatics and Biomedicine (BIBM).

[40] Li Y., Kang C., Qu X., Zhou Y., Wang W., Hu Y. (2016). Depression-Related Brain Connectivity Analyzed by EEG Event-Related Phase Synchrony Measure. Front. Hum. Neurosci..

[41] Castelnovo A., Casetta C., Cavallotti S., Marcatili M., Del Fabro L., Canevini M.P., Sarasso S., D’Agostino A. (2024). Proof–of–concept evidence for high–density EEG investigation of sleep slow wave traveling in First–Episode Psychosis. Sci. Rep..

[42] Hindriks R., van Putten M.J.A.M., Deco G. (2014). Intra-Cortical Propagation of EEG Alpha Oscillations. Neuroimage.

[43] Muller L., Chavane F., Reynolds J., Sejnowski T.J. (2018). Cortical Travelling Waves: Mechanisms and Computational Principles. Nat. Rev. Neurosci..

[44] Sinha S.R., Sullivan L.R., Sabau D., Orta D.S.J., Dombrowski K.E., Halford J.J., Hani A.J., Drislane F.W., Stecker M.M. (2016). American Clinical Neurophysiology Society Guideline 1: Minimum Technical Requirements for Performing Clinical Electroencephalography. Neurodiagn. J..

[45] Muller L., Reynaud A., Chavane F., Destexhe A. (2014). The Stimulus-Evoked Population Response in Visual Cortex of Awake Monkey Is a Propagating Wave. Nat. Commun..

[46] Burkitt G.R., Silberstein R.B., Cadusch P.J., Wood A.W. (2000). Steady-State Visual Evoked Potentials and Travelling Waves. Clin. Neurophysiol..

[47] Hallatschek O., Geyrhofer L. (2016). Collective Fluctuations in the Dynamics of Adaptation and Other Traveling Waves. Genetics.

[48] Sato N. (2022). Cortical Traveling Waves Reflect State-Dependent Hierarchical Sequencing of Local Regions in the Human Connectome Network. Sci. Rep..

[49] Gilbert J.R., Wusinich C., Zarate C.A. (2022). A Predictive Coding Framework for Understanding Major Depression. Front. Hum. Neurosci..

[50] Haarsma J., Kok P., Browning M. (2020). The Promise of Layer-Specific Neuroimaging for Testing Predictive Coding Theories of Psychosis. Schizophr. Res..

[51] Friston K.J. (2019). Waves of Prediction. PLoS Biol..

[52] Adams R.A., Stephan K.E., Brown H.R., Frith C.D., Friston K.J. (2013). The Computational Anatomy of Psychosis. Front. Psychiatry.

[53] Smith R., Badcock P., Friston K.J. (2020). Recent Advances in the Application of Predictive Coding and Active Inference Models within Clinical Neuroscience. Psychiatry Clin. Neurosci..

[54] Massimini M., Huber R., Ferrarelli F., Hill S.L., Tononi G. (2004). The Sleep Slow Oscillation as a Traveling Wave. J. Neurosci..

[55] Lozano-Soldevilla D., VanRullen R. (2019). The Hidden Spatial Dimension of Alpha: 10-Hz Perceptual Echoes Propagate as Periodic Traveling Waves in the Human Brain. Cell Rep..

[56] Bahramisharif A., van Gerven M.A.J., Aarnoutse E.J., Mercier M.R., Schwartz T.H., Foxe J.J., Ramsey N.F., Jensen O. (2013). Propagating Neocortical Gamma Bursts Are Coordinated by Traveling Alpha Waves. J. Neurosci..

[57] VanRullen R., Macdonald J.S.P. (2012). Perceptual Echoes at 10 Hz in the Human Brain. Curr. Biol..

[58] Volk D., Dubinin I., Myasnikova A., Gutkin B., Nikulin V.V. (2018). Generalized Cross-Frequency Decomposition: A Method for the Extraction of Neuronal Components Coupled at Different Frequencies. Front. Neuroinform..

[59] De Rosa M., Ktori M., Vidal Y., Bottini R., Crepaldi D. (2022). Frequency-Based Neural Discrimination in Fast Periodic Visual Stimulation. Cortex.

